# Securing consensus in fractional-order multi-agent systems: Algebraic approaches against Byzantine attacks

**DOI:** 10.1016/j.heliyon.2024.e40335

**Published:** 2024-11-13

**Authors:** Yubin Zhong, Asad Khan, Muhammad Awais Javeed, Hassan Raza, Waqar Ul Hassan, Azmat Ullah Khan Niazi, Muhammad Usman Mehmood

**Affiliations:** a School of Mathematics and Information Science, Guangzhou University, Guangzhou 510006, PR China; b Metaverse Research Institute, School of Computer Science and Cyber Engineering, Guangzhou University, Guangzhou 510006, PR China; c School of Transportation, Southeast University, Nanjing, Jiangsu, 211189, PR China; d Department of Mathematics, School of Science, Mathematics and Technology, Wenzhou-Kean University, Wenzhou, PR China; e Department of Mathematics and Statistics, The University of Lahore, Sargodha 40100, Pakistan

**Keywords:** Fractional-order systems, Nonlinear dynamics, Byzantine attacks, Leader-following consensus, Fractional-order Lyapunov approach

## Abstract

This paper investigates the behavior of fractional-order nonlinear multi-agent systems subjected to Byzantine assaults, specifically focusing on the manipulations of both sensors and actuators. We employ weighted graphs, both directed and undirected, to illustrate the system's topology. Our methodology combines algebraic graph theory with fractional-order Lyapunov techniques to develop algebraic requirements for leader-following consensus, providing a robust framework for analyzing consensus dynamics in these complex systems. We present quantitative results demonstrating the effectiveness of our approach, including two numerical examples that validate the proposed requirements for consensus evaluation. Notably, our work highlights the novelty of using fractional-order systems to enhance resilience against adversarial conditions, contributing significantly to the field of multi-agent systems. By clarifying key terms and streamlining our language, we ensure accessibility for a broader audience while emphasizing the implications of our findings for real-world applications.

## 1 Introduction

Byzantine attacks pose significant threats as security measures in communication networks primarily focus on external risks. Nodes undergo group authentication (e.g., using WEP or WAP) and are trusted by fellow group members [1]. When a trusted node is compromised and acts maliciously to disrupt network communication, it's termed a Byzantine Attack. Such attacks have severe consequences for the network since other nodes typically rely solely on initial authentication [2]. Byzantine attacks can occur in various scenarios, such as on a college campus where students utilize peer-to-peer communication for file and lecture transfers. Despite passing multiple authentication stages (e.g., college network password, class-specific password, and collaboration group password), a rogue user can initiate a DoS attack by continuously flooding the channel with packets devoid of useful data ([3], [4]). Another example is a hostile battlefield environment, where an enemy captures a physical unit (e.g., a vehicle) and utilizes its wireless communication equipment to launch DoS attacks on other units. The main focus of safe localization research has traditionally been preventing attackers from manipulating reference data or guaranteeing trustworthy localization while under assault [5]. Many approaches have been put out in recent decades to deal with this issue. Using methods that protect reference data integrity and increase the robustness of the observation process is a simple starting point ([6], [7]). The goal of this method, known as the secure localization technique based on robust observations, is to safeguard the real-world qualities of beacon data. Prominent instances of using time, space, or signal coding methods are the SeRLoc algorithm [8] and the distance bounding protocol ([9], [10]). These techniques might not be appropriate for large-scale mass adoption, though, as they frequently call for extra hardware components [11]. In situations where attackers would unavoidably introduce altered observations—also known as malicious observations—researchers have suggested tactics centered around the identification and removal of those harmful observations ([12], [13]). The remaining truthful observations are then used for node localization [14]. The MEF-based localization method is an example of an algorithm that uses this technique, which is sometimes known as the hostile node detection-based secure adaptation technique [15]. Federated learning (FL) has become a revolutionary computer paradigm ([16], [17]) to address these issues. With FL, users may work together to calculate a worldwide machine learning model while keeping their local data secret. To ensure that users' private data stays on their devices, FL distributes the model learning process to end users, such as intelligent devices, aggregating user-specific local models to create a global model. This method improves user privacy while greatly reducing bandwidth costs [18]. Recent research, however, has brought attention to FL's vulnerability to Byzantine assaults, in which malevolent users might tamper with gradients or actual models to impede learning, or contaminate training data to cause false positives in the global model. Blanchard et al. [19] showed that training convergence can be threatened and the global model's performance can be negatively impacted by a single hostile user. Many protection techniques against Byzantine assaults have been put out in an effort to lessen this problem ([20], [21]). Even while these studies have been successful in the short term in preventing Byzantine assaults, it is still difficult to fully defend FL [22]. It is a challenging task to protect FL from Byzantine assaults while attending to issues of efficiency, privacy, and data delivery, especially when its conventional procedures contain unidentified attack surfaces. The security and privacy aspects of federated learning were thoroughly reviewed in a recent survey conducted by Mothukuri et al. [23]. But it didn't test new theories to bolster its conclusions for more study, nor did it carry out tests to concurrently assess pre-existing schemes—an essential step for equitable comparisons. Network intrusion detection systems, or NIDSs, are an essential part of the tool used to protect computer infrastructures from hostile activity ([24], [25]). NIDSs are made expressly to keep an eye on computer networks and use network traffic analysis to find harmful intents. In today's large computer networks, which are made up of several sub-networks, traffic monitoring frequently requires a separated strategy. Placed strategically at different points, sensors and traffic analysis modules are each in charge of keeping an eye on and analyzing the flow of traffic inside a particular sub-network [26]. Normally, NIDS monitoring modules are networked together to facilitate the exchange of security data gathered from various sub-networks. The NIDS organization can be classified as dispersed, hierarchical, or centralized based on how this information is communicated ([27], [28]). Monitoring modules, which may just gather data, send that data up the hierarchy for additional analysis in centralized and hierarchical NIDS designs. Nevertheless, these systems are susceptible to assaults that target the more advanced modules, which might disable the system as a whole. Distributed intrusion detection systems ([29], [30] are used to reduce these risks by preventing single points of failure. Detection and analysis modules in distributed intrusion detection systems frequently serve as all-inclusive sensors [31]. In order to counter assaults from several sources at the same time, including distributed denial of service attacks, they cooperate together to organize coordinated responses throughout the network or improve the detection precision of each NIDS module. Data needed to be delivered to a central location for additional processing in early distributed systems ([32], [33]), which were built on a master-slave architecture. However, modern methods make use of peer-to-peer networks ([34], [35]), which make it possible to identify assaults via completely distributed examination of shared data. Our present effort is significantly related to research investigations that concentrate on the identification of Byzantine nodes. A consensus-based spectrum sensing algorithm for ad hoc wireless networks includes an approach that uses outlier detection techniques to identify compromised nodes ([36], [37]). The attack models that are used to undermine the spectrum sensing technique are further examined in Reference [39]. One such model is a covert adaptive data injection assault, in which findings from sensing are used to dynamically change attack techniques. Isolating neighbor nodes that show notable variances in their numerical data from a predetermined norm is the suggested protection mechanism against such assaults [39]. Reputation-based trust management techniques are used to handle the discovery of Byzantine nodes. In particular, this work addresses attacks in which malicious robots leak incorrect data to nearby nodes in a consensus-based multi-robot system, especially for formation control.

Most existing studies have focused on integer-order systems, which are limited in their ability to account for the long-term memory and hereditary dynamics often present in real-world systems. Integer-order models typically assume that system behavior at any given time depends only on the current state, which may not be sufficient for capturing complex dynamics in adversarial environments, such as those influenced by Byzantine attacks. These attacks introduce uncertainties and disruptions that require systems to not only respond to present conditions but also account for past interactions and accumulated effects. Fractional-order systems, in contrast, offer a more flexible modeling approach due to their inherent ability to capture both present and past dynamics through fractional derivatives. These systems retain memory of previous states, allowing for a more accurate representation of processes that involve long-range dependence or gradual changes over time. This memory effect is particularly beneficial when dealing with Byzantine attacks, as it enables the system to better mitigate the impact of malicious agents by taking into account the history of interactions, thus providing enhanced resilience. By modeling multi-agent systems with fractional-order dynamics, our approach allows for a more robust response to malicious behaviors. The inclusion of memory effects enhances the system's ability to filter out incorrect information injected by compromised agents, ultimately improving both the stability and accuracy of consensus. This novel framework addresses a critical gap in the literature, providing a new direction for improving the resilience of consensus protocols in adversarial settings. Consequently, our contribution significantly advances the state of the art in fractional-order multi-agent systems by offering more sophisticated tools for achieving reliable consensus under Byzantine attacks.

Motivated by our previous discussion, we outlined contributions in a way that:

In this paper, we contribute to the field by studying fractional-order nonlinear systems under Byzantine attacks, including sensor and actuator attacks, within the framework of weighted directed graphs. Notably, our research addresses a significant gap in the literature concerning consensus in fractional-order multi-agent systems under Byzantine attacks. To fill this gap, we propose a novel approach utilizing fractional-order Lyapunov methods. Our contribution includes the selection of a simple quadratic Lyapunov function and the development of algebraic criteria for leader-following consensus using graph theory and linear matrix inequalities. These criteria offer a practical and efficient means of evaluating consensus in such systems. Furthermore, our results extend to fractional-order multi-agent systems with undirected topologies. Finally, we provide two illustrative examples to demonstrate the effectiveness of our proposed methodology.

Our work advances the state of the art by introducing a novel application of fractional-order Lyapunov methods to address consensus under Byzantine attacks, which has been minimally explored. Furthermore, the proposed algebraic criteria offer a more practical and efficient approach for consensus evaluation in both directed and undirected multi-agent systems, filling a critical gap in the current literature.

The structure of the remaining content is as follows:

1. In the 1 section, we introduce the impacts of Byzantine attacks on the controller and explore them from various perspectives.
2. In the section 2 graph theory explained for the communication of agents, some lemmas and definitions provided.
3. In the section 3 provided a problem formation, effects of attack on MAS, some definitions and lemmas provided and theorem proved for the proof of theoretical results.
4. In the section 4 Provided numerical experiments, and two examples provided for the effectiveness of results.
5. In section 5 made conclusions

**Notation.** Given that R represents the set of real numbers, $R^{n}$ and $R^{n_{1}\times n_{2}}$ indicate the n-dimensional real vector space and $n_{1}\times n_{2}$ real matrices, respectively. Here, $I_{n}$ stands for the n-dimensional identity matrix, and “T” signifies matrix transposition. The notation $\lambda_{max}(S)$ denotes the maximum eigenvalue of matrix *S*, where *S* is a real matrix. For a vector $z\in R^{n}$, its norm is described as $|z|=\sqrt{z^{T}z}$.

## 2 Preliminaries

### 2.1 Communication typologies

Let G = (V, E) be a directed weighted graph, which consists of a set of nodes $V=\{v_{1},v_{2},...,v_{N}\}$ and a set of directed edges $E\subset \{(v_{i},v_{j}):v_{i},v_{j}\in V\}$. Each directed edge $\left(v_{i},v_{j}\right)$ denotes an edge starting at node $v_{i}$ and ending at node $v_{j}$, where $v_{i}$ and $v_{j}$ are referred to as the tail and head, respectively. $N_{i}=\{v_{j}\;|\;(v_{j},v_{i})\in E\}$ denotes the set of neighbors of node $v_{i}$. A weighted adjacency matrix $A={\left(a_{ij}\right)}_{N\times N}$, where $a_{ij}>0$ if $(v_{i},v_{j})\in E$, else $a_{ij}=0$. A directed spanning tree of the digraph *G* is a sub-graph of *G* where the directed edges allow the root node to reach every other node.

The Laplacian matrix $L={\left(l_{ij}\right)}_{N\times N}$ of graph *G* is defined as follows:

$$l_{ij}=\left\{ \begin{array}{cc} -a_{ij}, & i\neq j\\ \sum_{\begin{array}{c} k=1\\ k\neq i \end{array}}^{N}a_{ik}, & i=j. \end{array}\right.$$

It is evident that $\sum_{\begin{array}{c} j=1 \end{array}}^{N}l_{ij}=0$ for i=1,2,...,N.

Lemma 2.1 *[30]* *Inequality hold for the matrices L, M, N*

$$\left(\begin{array}{cc} L & M\\ M^{T} & N \end{array}\right)<0.$$

*Which also hold the* $(N-M^{T}L^{-1}M)<0\;and\;L<0$ *.*

Lemma 2.2 *[29]* *Given matrices* $L\in R^{n\times n}$ *and* $M\in R^{r\times r}$ *with eigenvalues* $\zeta_{1},\zeta_{2},\ldots ,\zeta_{n}$ *and* $\psi_{1},\psi_{2},\ldots ,\psi_{r}$ *respectively, the eigenvalues of their Kronecker product* L⊗M *can be expressed as* $\zeta_{i}\psi_{j}$ *for* i=1,2,…,n *and* j=1,2,…,r *.*

Lemma 2.3 *[28]* *Suppose that* $u[k]\in R^{n}$ *and* $z_{i}[k]\in R^{n}$ *(where* $z_{i}[k]$ *represents the state of agents) are discrete functions. Then the following relationship holds:*

$$\nabla_{T}^{\alpha }(u^{T}[k]u[k])\leq 2u^{T}[k]\;\nabla_{T}^{\alpha }u[k]\;\forall \alpha \in (0,1),$$

*where* $\nabla_{T}^{\alpha }$ *is the discrete-time fractional difference operator.*

*We now consider a general discrete fractional nonlinear equation with time delay:*

$$\nabla_{T}^{\alpha }z_{i}[k]=f(k,z_{k})\;k\geq k_{0},$$

*where* 0<α≤1 *,* $z_{k}[\xi ]=z_{i}[k+\xi ]$ *for* ξ∈{−r,…,0} *, and f maps* R× *(bounded sets of* **N** *) into bounded sets of* $R^{n}$ *, satisfying* f(k,0)=0 *.*

Definition 2.1 [32] A function $f:R^{r}\times R\to R^{r}$ is said to be QUAD(Δ,η) if there exists a positive constant *η* and a diagonal matrix $\Delta \in R^{r\times r}$ such that:

$${\left(m-n\right)}^{T}[f(k,m)-f(k,n)]-{\left(m-n\right)}^{T}\Delta (m-n)\leq -\eta {\left(m-n\right)}^{T}(m-n),$$

for any $m,n\in R^{r}$.

Definition 2.2 [38] The discrete-time difference operator of order *α* for a function f(k) is defined as:

$$\nabla_{T}^{\alpha }f(k)=\frac{1}{T^{\alpha }}\sum_{r=0}^{\left\lfloor \frac{k}{T}\right\rfloor }{\left(-1\right)}^{r}\left(\begin{array}{c} \alpha \\ r \end{array}\right)f(k-rT),\;\alpha >0,$$

where T is the time step. For convenience, when T = 1, this reduces to:

$$\nabla^{\alpha }f(k)=\sum_{r=0}^{k}{\left(-1\right)}^{r}\left(\begin{array}{c} \alpha \\ r \end{array}\right)f(k-r).$$

## 3 Problem formation

The paper explores the complexity of Byzantine attacks, including weaknesses related to both actuators and sensors, by examining a leader-following consensus framework for fractional-order nonlinear multi-agent systems. The paper provides brief conditions based on fractional-order concepts to strengthen consensus dynamics through a careful investigation. These settings are designed to foster positive agent interaction while strengthening resistance to harmful interference. The research provides useful tactics for enhancing system security and resilience in complex operating situations by examining fractional-order dynamics in combination with several attack vectors.

Consider a MAS consists of n agents, then the dynamics of follower i-th agents can be described as.

(1) $$\nabla_{T}^{\alpha }z_{i}(k+1)=Mz_{i}(k)+h(k,z_{i}(k))+(u_{i}(k)+g_{i}(k)).$$

Where $z_{i}(k)$, $u_{i}(k)$, $h(k,z_{i}(k))$, and $g_{i}(k)$ show the state of follower agent, control input, nonlinear function, and attack signals on the follower agent respectively. M is a constant matrix.

Similarly, the dynamics of leader agents.

(2) $$\nabla_{T}^{\alpha }z_{0}(k+1)=Mz_{0}(k)+h(k,z_{0}(k))+u_{0}(k).$$

Where $z_{0}(k)$, $u_{0}(k)$ and $h(k,z_{0}(k))$ denote the state of the leader agent, control input, and nonlinear function respectively.

### 3.1 Effect of attacks on MAS

The MAS under the actuator attack can be represented as

(3) $$u_{i}^{\overset{˜}{c}}(k)=u_{i}(k)+\lambda_{i}u_{i}^{b}(k).$$

Where $u_{i}(k)$, $u_{i}^{c}(k)$ and $u_{i}^{b}(k)$ represents the nominal state, corrupted control input and attack signals. The attack occurs only if $\lambda_{i}$=1; otherwise $\lambda_{0}$=0.

The MAS under the sensor attack which can be expressed as

(4) $$z_{i}^{c}(k)=z_{i}(k)+\gamma_{i}z_{i}^{b}(k).$$

Where $z_{i}^{c}(k)$, $z_{i}(k)$, $z_{i}^{b}(k)$ shows the corrupted input, nominal state and attack signal. Similarly attack only when if $\gamma_{i}$=1 otherwise $\gamma_{i}$=0.

Now combining the effect of both attacks Eq (3) and Eq (4) we can write such that.

(5) $$g_{i}(k)=\lambda u_{i}^{b}(k)+d_{k}\sum_{m_{i}}a_{ij}[\gamma_{j}z_{j}^{b}(k)-\gamma_{i}z_{i}^{b}(k)].$$

Where actuator attack and sensor attack are represented by $z_{i}^{b}(k)$ and $u_{i}^{b}(k)$, respectively, $z_{j}^{b}(k)$ represents the attack signals with nearby j of agent i. Where $d_{k}$ and $a_{ij}$ represent the scaler gain, and (i, j) adjacency matrix A.

Now design a control protocol under the effect of both attacks combining Eq (5).

(6) $$u_{i}(k)=K\sum_{j=1}^{N}a_{ij}(z_{j}(k)-z_{i}(k))+Ka_{i0}(z_{0}(k))+\lambda_{i}u_{i}^{b}(k)+d_{k}\sum_{j=1}^{N}a_{ij}(\gamma_{j}z_{j}^{b}(k)-\gamma_{i}z_{i}^{b}(k)).$$

**Figure 1:**
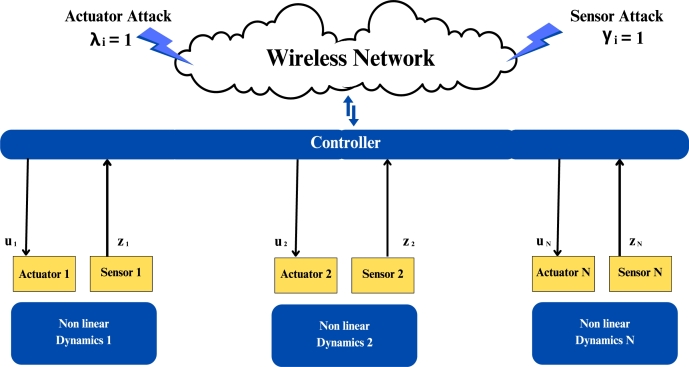
Network communication topology of nonlinear with directed connected graph agents under the sensor attack and actuator attack.

Where $u_{i}(k)$ is the control input for the *i*-th follower agent. *K* is Controller gain. $a_{ij}$ is the adjacency matrix element representing connectivity between agents, $x_{j}(k)$ represents the state of the *j*-th agent at time *k*, $x_{0}(k)$ also represents the state of the leader agent at time *k*. $\lambda_{i}$ indicates actuator attack occurrence on the *i*-th follower agent, $u_{i}^{b}(k)$ actuator attack signal, $d_{k}$ is scalar gain for sensor attack compensation, $\gamma_{j}$ also indicates sensor attack occurrence on the *j*-th follower agent, $z_{j}^{b}(k)$ is sensor attack signal for the *j*-th follower agent. $\gamma_{i}$ also indicator of sensor attack occurrence on the *i*-th follower agent and $z_{i}^{b}(k)$ is sensor attack signal for the *i*-th follower agent. Nonlinear with directed connected graph agents under the sensor attack and actuator attack shown in Fig. 1.

Definition 3.1 [27] The follower-leader Eq (1)-(2) consensus converges to zero under the control protocol Eq (6) when i=1,2,3,..., N.

(7) $$\lim_{k\to \infty }\|z_{i}(t)-z_{0}(t)\|=0.$$

So, the error $\lim_{k\to \infty }\|\sigma_{i}(k)\|=0$.

We make some lemmas and hypothesis:

$(H_{1})$ for QUAD g(z) is both quadratic in its argument and its derivative is bounded by a quadratic function of the argument, expressed as $|g(z)|\leq \eta |z{|}^{2}$ and $\left|g^{\prime }(z)|\leq \eta |z\right|$, respectively.

$(H_{2})$. The leader is rooted in a spanning tree contained in the corresponding digraph of the multi-agent system.

Remark 3.1 A positive real component of the eigenvalues of the matrix H⊗K indicates a link between the square of matrix *H* and the positively of real sections of eigenvalues if the condition $\left(H_{2}\right)$ in Lemma 2.2 is met. This need is necessary in order to ensure convergence or stability.

Theorem 3.2 *The leader-following consensus of system Eq* (1) *and Eq* (2) *under the control law Eq* (6) *may be reached as long as* $\left(H_{1}\right)$ *and* $\left(H_{2}\right)$ *hold, and there exist a scalar* Q>0 *and a positive definite matrix* S>0 *such that,*

(8) $$I_{N}⊗(M+M^{T}+2\Delta -2\eta I_{n}+\beta I_{n}+S)+\frac{1}{\beta }(H^{T}⊗K^{T})(H⊗K)<0$$

*and*

(9) $$I_{N}⊗(\beta I_{n}-S)+\frac{1}{\beta }(I_{N}⊗N^{T})(I_{N}⊗N)<0.$$

Proof We take $\sigma_{i}(k)=z_{i}(k)-z_{0}(k)$, for i=1,2,…,N. As the leader-following consensus requires that $\sigma_{i}(k)\to 0$ as k→∞, which aligns with the convergence requirement stated in Eq (7), $\lim_{k\to \infty }\|z_{i}(t)-z_{0}(t)\|=\lim_{k\to \infty }\|\sigma_{i}(k)\|=0$.

Subtracting Eq (1) from Eq (6) and using Eq (5), we get

(10) $$\nabla_{T}^{\alpha }\sigma (k)=M\sigma_{i}(k)+h(k,z_{i}(k))-h(k,z_{0}(k))\;+K\sum_{j=1}^{N}a_{ij}(\sigma_{j}(k)-\sigma_{i}(k))+Ka_{i0}(\sigma_{0}(k))+\lambda_{i}u_{i}^{b}(k)\;+d_{k}\sum_{j=1}^{N}a_{ij}(\gamma_{j}\sigma_{j}^{b}(k)-\gamma_{i}\sigma_{i}^{b}(k)).$$

Choose the Lyapunov function in quadratic form,

$$\upsilon (k)=\sum_{i=1}^{N}\sigma_{i}^{T}(k)\sigma_{i}(k).$$

Taking the derivative of order *α* of the Lyapunov function, using Lemma 2.3, and solving Eq (10),

$$\nabla_{T}^{\alpha }\upsilon (k)\leq 2\sum_{i=1}^{N}\sigma_{i}(k)\nabla_{T}^{\alpha }\sigma_{i}(k)$$

(11) $$=2\sum_{i=1}^{N}\sigma_{i}^{T}(k)[M\sigma_{i}(k)+h(k,z_{i}(k))-h(k,z_{0}(k))\;+K\sum_{j=1}^{N}a_{ij}(\sigma_{j}(k)-\sigma_{i}(k))+Ka_{i0}(\sigma_{0}(k))+\lambda_{i}u_{i}^{b}(k)\;+d_{k}\sum_{j=1}^{N}a_{ij}(\gamma_{j}\sigma_{j}^{b}(k)-\gamma_{i}\sigma_{i}^{b}(k))].$$

By using the Definition 2.1 and satisfying the hypothesis,

(12) $$\sigma_{i}^{T}(k)[h(k,z_{i}(k))-h(k,z_{0}(k))]\leq \sigma_{i}^{T}(k)(\Delta -\eta I_{n})\sigma_{i}(k).$$

By using the Eq (12), we can write the Eq (11)

$$\nabla_{T}^{\alpha }\sigma_{i}(k)\leq 2\sum_{i=1}^{N}\sigma_{i}^{T}(k)(M+\Delta -\eta I_{n})\sigma_{i}(k)\;+2d_{k}\sum_{i=1}^{N}\sigma_{i}(I_{N}⊗N)\sum_{j=1}^{N}\gamma_{j}\sigma_{j}^{b}u_{i}^{b}(k)\;+\lambda_{i}u_{i}^{b}(k)-2\sum_{i=1}^{N}\sigma_{i}^{T}(k)\sum_{j=1}^{N}l_{ij}\sigma_{j}(k)-2\sum_{i=1}^{N}a_{i0}\sigma_{i}^{T}(k)K\sigma_{i}(k).$$

$$\nabla_{T}^{\alpha }\upsilon (k)\leq 2\sum_{i=1}^{N}\sigma_{i}^{T}(k)(M+\Delta -\eta I_{n}+L)\sigma_{i}(k)\;-2\sigma^{T}(k)(I_{N}⊗L)\sigma (k)-2\sum_{i=1}^{N}a_{i0}\sigma_{i}^{T}(k)K\sigma_{i}(k).$$

To satisfy equation (8), using Lemma 2.3, rewrite in the form,

$$\sigma^{T}(k)\left[2(M+M^{T}+2\Delta -2\eta I_{n}+L)⊗I_{n}\right]\sigma (k).$$

Thus, to satisfy equation (8),

$$2(M+M^{T}+2\Delta -2\eta I_{n}+L)⊗I_{n}+\frac{1}{\beta }(H^{T}⊗K^{T})(H⊗K)<0.$$

For equation (9), rewrite involving *K* as,

$$-2\sigma^{T}(k)(I_{N}⊗(\beta I_{n}-S))\sigma (k).$$

Therefore, to satisfy equation (9),

$$I_{N}⊗(\beta I_{n}-S)+\frac{1}{\beta }(I_{N}⊗N^{T})(I_{N}⊗N)<0.$$

From Lemma 2.3 and equations (8) and Eq (9), we conclude that,

$$\left(\begin{array}{cc} I_{n}⊗(M+M^{T}+2\Delta +\beta I_{n}+S) & -H⊗K\\ -H^{T}⊗K^{T} & -\beta I_{n}⊗I_{n} \end{array}\right)<0$$

and

$$\left(\begin{array}{cc} I_{N}⊗(\beta I_{n}-S) & I_{n}⊗N\\ I_{N}⊗N^{T} & -\beta I_{N}⊗I_{n} \end{array}\right)<0.$$

Thus, we conclude that equation that the system asymptotically stable and the consensus of the system is achieved. □

## 4 Numerical experiments

In the numerical experiments, we provide two examples for the effectiveness and prove above theoretical result.

**Figure 2:**
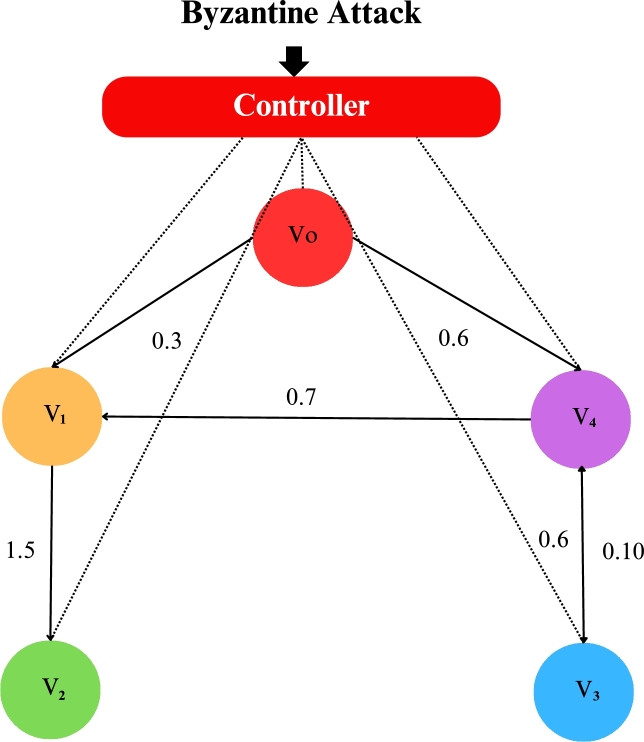
Network communication topology of nonlinear with directed connected graph agents under the byzantine attack.

**Figure 3:**
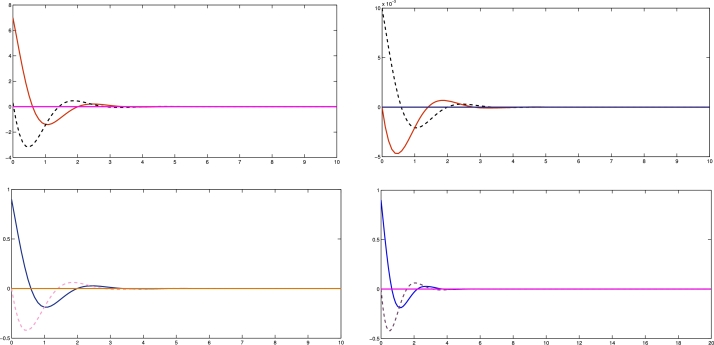
State error trajectories of leader-following MAS.

**Figure 4:**
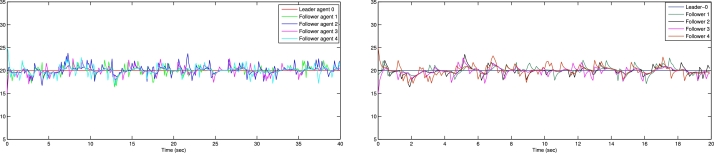
Show the influence of sensor and actuator attack on leader and four follower multi agent system.

**Figure 5:**
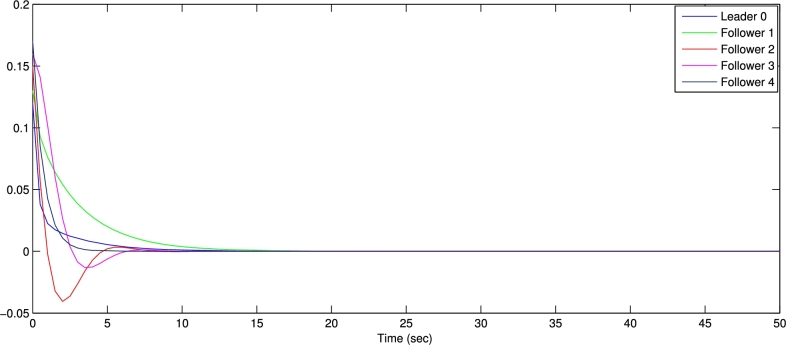
Shows the impact of both attacks on control protocol performance.

Example 4.1 We consider that there is leader and four follower MAS which shown in Fig. 2. The error trajectories of MAS shown in Fig. 3. The Fig. 4 shows the influence of sensor and actuator attacks on one leader and four follower multi-agents and the Fig. 5 shows the impact of both attacks on control protocol performance. Let us define the matrices M and N such that

$$M=\left(\begin{array}{ccc} -4.4 & 0 & 1.2\\ 0 & -4.3 & 0\\ 0.9 & 0 & -4.6 \end{array}\right),and\;N=\left(\begin{array}{ccc} 0.3 & 0 & 0\\ 0.2 & 0.4 & 0\\ 0 & 0 & 0.16 \end{array}\right).$$

We define the network communication according to Fig. 2,

$$A=\left(\begin{array}{cccc} 0 & 0 & 0 & 0.7\\ 1.5 & 0 & 0 & 0\\ 0 & 0 & 0 & 0.6\\ 0 & 0 & 0.10 & 0 \end{array}\right),and\;A_{0}=\left(\begin{array}{cccc} 0.3 & 0 & 0 & 0\\ 0 & 0 & 0 & 0\\ 0 & 0 & 0 & 0\\ 0 & 0 & 0 & 0.6 \end{array}\right).$$

Similarly, we can write the Laplacian matrix L and H, we get it as follows $L=H=L+A_{0}$ such that,

$$L=\left(\begin{array}{cccc} 0.7 & 0 & 0 & 0.7\\ -1.5 & 1.5 & 0 & 0\\ 0 & 0 & 0.6 & -0.6\\ 0 & 0 & -0.10 & 1.5 \end{array}\right),and\;H=\left(\begin{array}{cccc} 0.9 & 0 & 0 & -0.7\\ -1.5 & -1.5 & 0 & 0\\ 0 & 0 & 0.6 & -0.5\\ 0 & 0 & -0.10 & 1.5 \end{array}\right).$$

We define η=0.6, α=0.9, $\Delta =\left(\begin{array}{ccc} 0.7 & 0 & 0\\ 0 & 0.7 & 0\\ 0 & 0 & 0.7 \end{array}\right)$, and $h(k,z_{i}(k))=\frac{1}{6}sinz_{i}(k)$.

Given,

• Actuator attack signal $u_{i}^{b}(k)=0.5$.
• Actuator attack indicator $\lambda_{i}$ = 1 (attack occurs).
• Sensor attack signal $z_{i}^{b}(k)=0.3$.
• Sensor attack indicator $\gamma_{i}$ = 1 (attack occurs).
• Scalar gain for sensor attack compensation $d_{k}=0.2$.
• Adjacency matrix A as provided earlier,

The expression for $g_{i}(k)$ is,

$$g_{i}(k)=\lambda_{i}u_{i}^{b}(k)+d_{k}\sum_{j=1}^{N}a_{ij}[\gamma_{j}z_{j}^{b}(k)-\gamma_{i}z_{i}^{b}(k)].$$

Substituting the given numerical values and the adjacency matrix,

$$g_{i}(k)=1\times 0.5+0.2\times \left(0\times (0.3-0.3)+1.5\times (0.3-0.3)+0\times (0.3-0.3)+0.7\times (0.3-0.3)\right)=0.5+0=0.5.$$

Where $\beta =0.6,S=\left(\begin{array}{ccc} 0.8 & 0 & 0.09\\ 0 & 0.6 & 0\\ 0.09 & 0 & 0.7 \end{array}\right)$ and K=$\left(\begin{array}{ccc} 0.3 & 0 & 0\\ 0.4 & 0.5 & 0\\ 0 & 0 & 0.2 \end{array}\right)$.

**Figure 6:**
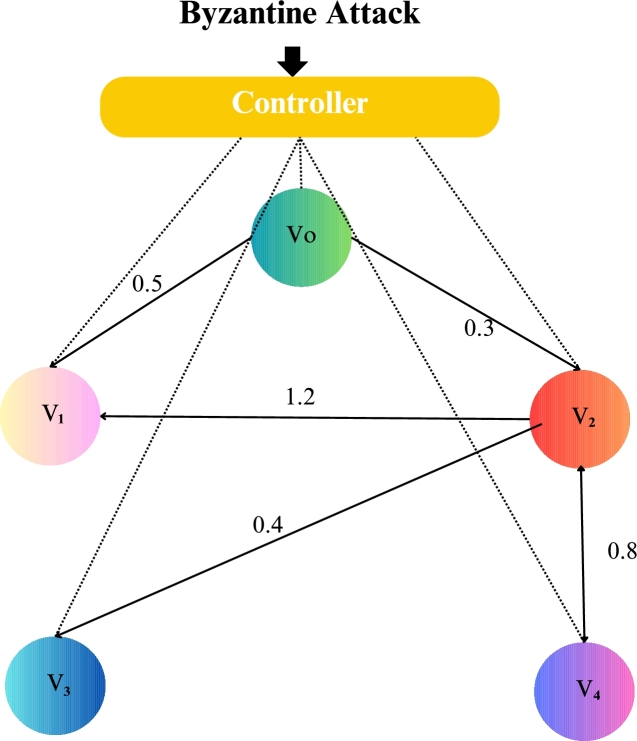
Network communication topology of nonlinear of agents connected through an undirected graph under the byzantine attack.

**Figure 7:**
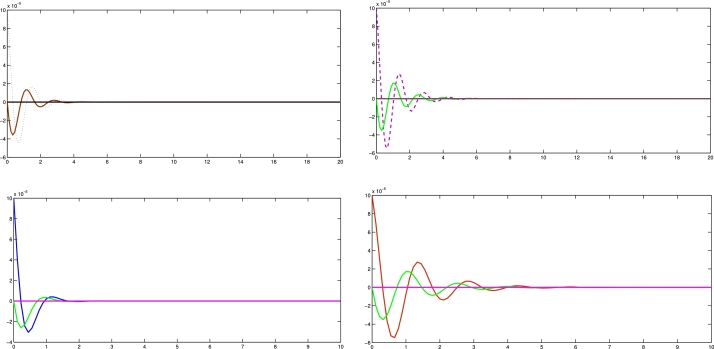
State error trajectories of leader-following MAS.

**Figure 8:**
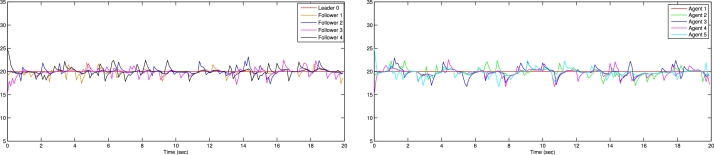
Show the influence of sensor attack and actuator attacks on one leader and four follower agents.

**Figure 9:**
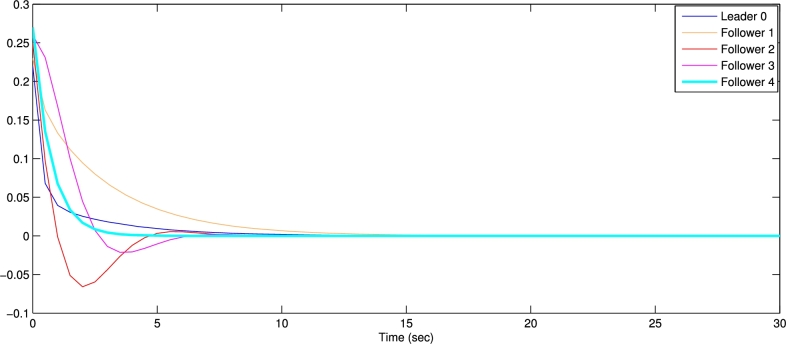
Shows the effect of attacks on control protocol performance.

Example 4.2 Similarly, we consider a leader and four followers which are shown in Fig. 6 and state error trajectories shown in Fig. 7. The Fig. 8 shows the influence of sensor and actuator attack on one leader and four follower multi-agent system. And Fig. 9 shows the effect of both attacks on control protocol performance.

Similarly we define the matrices M and N such that

$$M=\left(\begin{array}{ccc} -3.9 & 0 & 0.6\\ 0 & -4.6 & 0\\ 2 & 0 & -3.8 \end{array}\right),and\;N=\left(\begin{array}{ccc} 0.3 & 0 & 0\\ 0.3 & 0.4 & \\ 0 & 0 & 0.2 \end{array}\right).$$

$$A=\left(\begin{array}{cccc} 0 & 1.2 & 0 & 0\\ 1.2 & 0 & 0.4 & 0.8\\ 0 & 0.4 & 0 & 0\\ 0 & 0.8 & 0 & 0 \end{array}\right),and\;A_{0}=\left(\begin{array}{cccc} 0.5 & 0 & 0 & 0\\ 0 & 0.3 & 0 & 0\\ 0 & 0 & 0 & 0\\ 0 & 0 & 0 & 0 \end{array}\right).$$

Similarly, we can derive the laplacian matrices as we calculate in Example 4.1.

$$L=\left(\begin{array}{cccc} 1.2 & -1.2 & 0 & 0\\ -1.2 & 2.2 & -0.4 & -0.8\\ 0 & -0.4 & 0.4 & 0\\ 0 & -0.8 & 0 & 0.8 \end{array}\right),and\;H=\left(\begin{array}{cccc} 1.6 & -1.2 & 0 & 0\\ -1.2 & 2.4 & -0.4 & -0.8\\ 0 & -0.4 & 0.4 & 0\\ 0 & -0.8 & 0 & 0.8 \end{array}\right).$$

Where we define $h(k,z_{i}(k))=\frac{1}{2}tanhz_{i}(k)$, $\Delta =\left(\begin{array}{ccc} 0.6 & 0 & 0\\ 0 & 0.6 & 0\\ 0 & 0 & 0.6 \end{array}\right)$, η=0.27, $S=\left(\begin{array}{ccc} 0.5 & 0 & 0.5\\ 0 & 0.6 & 0\\ 0.05 & 0 & 0.6 \end{array}\right)$

and K=$\left(\begin{array}{ccc} 0.4 & 0 & 0\\ 0.3 & 0.6 & 0\\ 0 & 0 & 0.5 \end{array}\right)$. Remaining values remain same as mentioned in Example 4.1.

### 4.1 Explanation

The figures in this paper visually represent the dynamics of leader-following multi-agent systems (MAS) under various attack conditions, including sensor, actuator, and Byzantine attacks. Figure 1, Figure 2 illustrate the network communication typologies of nonlinear agents using directed graphs, showcasing the system's vulnerability when subject to sensor, actuator, and Byzantine assaults. Figure 3, Figure 4, Figure 7 plot the state error trajectories of the leader and follower agents, emphasizing how the system converges to consensus or deviates when attacks occur. Figure 5, Figure 8 delve deeper into the impact of these attacks on the control protocol, highlighting the system's robustness under different attack profiles. Annotating key transitions and deviations across these figures offer a clearer understanding of how the control strategies perform, enhancing the visual presentation and ensuring that critical behaviors are evident to the reader. Fig. 6 depicts the network communication topology of nonlinear agents connected through an undirected graph under Byzantine attack, offering insight into how different communication structures influence system resilience. Fig. 9 summarizes the effect of various attacks on the control protocol performance, showing how these adversarial conditions degrade or influence the system's control and stability.

### 4.2 Camparsion with existing results

In this paper, we propose a novel approach for achieving leader-following consensus in fractional-order multi-agent systems under Byzantine attacks, drawing comparisons with existing consensus methodologies in the literature. For example, the study [40] highlights the importance of integrating multiple renewable energy sources for enhanced reliability. Similarly, our approach leverages fractional-order dynamics to address the complexities introduced by adversarial conditions, demonstrating superior resilience compared to traditional integer-order systems. Additionally, research focused on fractional-order solutions for the nonlinear time-fractional [41] illustrates the application of advanced mathematical techniques in solving complex models. Our use of the fractional-order Lyapunov technique parallels this effort, providing deeper insights into consensus behavior amidst Byzantine attacks. Furthermore, the study of the [42] equation underscores innovative approaches in nonlinear systems, reinforcing the effectiveness of our method in modeling and enhancing system resilience against malicious behavior. By contrasting our work with these references, we underscore how our research advances the state of the art in consensus protocols, showcasing the practicality and robustness of our proposed framework in real-world applications where maintaining consensus is critical despite adversarial challenges.

### 4.3 Analysis and discussion

In our analysis and discussion, we emphasize the significance of the derived algebraic requirements for leader-following consensus in fractional-order multi-agent systems under Byzantine attacks. The step-by-step breakdown of the fractional-order Lyapunov technique reveals how the inclusion of memory effects enhances system resilience, allowing for more effective filtering of erroneous data from compromised agents. By utilizing both directed and undirected weighted graphs, we illustrate the complex interactions within the system and how these relationships influence consensus dynamics. The numerical examples provided serve to validate our theoretical framework, demonstrating that the proposed criteria not only hold under simulated conditions but also offer practical insights into the behavior of multi-agent systems facing adversarial conditions. This comprehensive discussion underscores the robustness of our approach and its potential applications in developing resilient consensus protocols in real-world scenarios.

## 5 Conclusion

Recent research has delved into fractional-order nonlinear multi-agent systems with leader-following consensus amidst byzantine attacks combining into sensor and actuator attack, yielding algebraic conditions via fractional-order Lyapunov methods. These conditions, formulated as linear matrix inequalities, offer a straightforward means to assess consensus. The examined multi-agent system aligns with a weighted directed graph, yet the derived conclusions extend to undirected graphs, showcasing the versatility of the proposed methodology across various multi-agent systems. Future endeavors will focus on exploring consensus within fractional-order singular multi-agent systems under Byzantine attacks, indicating a promising avenue for further investigation and advancement in decentralized control strategies.

## Code availability

The code is considered an intellectual property of the Guangzhou Government project, and therefore not publicly available.

## Funding

This research was sponsored by the 10.13039/501100001809National Natural Science Foundation of China grant No. 12250410247, and also by the Ministry of Science and Technology of China, grant No. WGXZ2023054L.

## CRediT authorship contribution statement

**Yubin Zhong:** Validation, Funding acquisition. **Asad Khan:** Data curation, Conceptualization. **Muhammad Awais Javeed:** Methodology, Investigation. **Hassan Raza:** Writing – original draft, Supervision. **Waqar Ul Hassan:** Resources, Data curation. **Azmat Ullah Khan Niazi:** Writing – review & editing, Validation, Project administration. **Muhammad Usman Mehmood:** Resources, Formal analysis.

## Declaration of Competing Interest

The authors declare that they have no known competing financial interests or personal relationships that could have appeared to influence the work reported in this paper.

Yubin Zhong and Asad Khan reports article publishing charges was provided by 10.13039/501100014881Guangzhou University. Yubin Zhong and Asad Khan reports a relationship with Guangzhou University that includes: employment and funding grants. The authors of this manuscript, declare that there are no conflicts of interest regarding the publication of this paper.
